# Risk factors for mortality in patients over 70 years old with COVID-19 in Wuhan at the early break: retrospective case series

**DOI:** 10.1186/s12879-021-06450-8

**Published:** 2021-08-16

**Authors:** Xu Zhu, Wenzheng Yuan, Junwei Shao, Kesheng Huang, Qingbo Wang, Shuang Yao, Wei Lu, Li Liu, Tao Fu

**Affiliations:** 1 Renmin Hospital, Wuhan University, Wuhan, People’s Republic of China; 2 School of Public Health, Tongji Medical College, Huazhong University of Science and Technology, Wuhan, People’s Republic of China

**Keywords:** COVID-19, Risk factor, Outcome, Clinical characteristics, Fatality rate

## Abstract

**Background:**

Elderly patients with COVID-19 were shown to have a high case-fatality rate. We aimed to explore the risk factors associated with death in patients over 70 years old (yr).

**Methods:**

In this retrospective study, we enrolled consecutively hospitalized patients over 70 yr with COVID-19 between January 20 and February 15, 2020 in Renmin Hospital of Wuhan University. Epidemiological, demographic, and clinical data were collected. Clinical subtypes, including mild, moderate, severe, and critical types, were used to evaluate the severity of disease. Patients were classified into two groups: survivor and non-survivor groups. Clinical data were compared between the two groups. Univariable and multivariable Cox regression methods were used to explore the risk factors.

**Results:**

A total of 147 patients were enrolled. The case-fatality rate was 28.6%. Multivariable Cox proportional hazard regression showed that clinical subtypes, including the severe type (HR = 2.983, 95% CI: 1.231–7.226, *P* = 0.016) and the critical type (HR = 3.267, 95%CI: 1.009–10.576, *P* = 0.048), were associated with increasing risk of death when compared with the general type. Blood urea nitrogen greater than 9.5 mmol/L (HR = 2.805, 95% CI: 1.141–6.892, *P* = 0.025) on admission was an independent risk factor for death among laboratory findings.

**Conclusion:**

The patients over 70 yr with COVID-19 had a high case-fatality rate. The risk factors, including clinical subtypes and blood urea nitrogen greater than 9.5 mmol/L, could help physicians to identify elderly patients with poor clinical outcomes at an early stage.

**Supplementary Information:**

The online version contains supplementary material available at 10.1186/s12879-021-06450-8.

## Background

The epidemic of 2019 novel coronavirus disease (COVID-19) was first reported in Wuhan, China [1–3]. Now, it has spread all over the world. In addition to severe acute respiratory syndrome coronavirus (SARS-CoV) and Middle East respiratory syndrome coronavirus (MERS-CoV), SARS-CoV-2 signified the third emergence of highly pathogenic coronavirus into the human. On February 11, 2020, novel coronavirus-infected pneumonia (NCIP) was named, and then “COVID-19” by the WHO [4]. As of October 9, 36,754,395 confirmed cases and 1,064,838 deaths were reported globally [5].

The clinical features of those patients include fever, nonproductive cough, dyspnea, myalgia, fatigue, diarrhea, normal or decreased leukocyte count, and imaging evidence of pneumonia. Severe organ dysfunction, including shock, acute respiratory distress syndrome (ARDS), acute heart injury, and acute kidney injury, can lead to death [6]. The outbreak has rapidly spread, and all ages can be easily infected [7]. Wang et al. reported that older males with comorbidities were more susceptible to COVID-19, and resulted in serious and life-threatening respiratory diseases [8]. To the best of our knowledge, few studies focused on characterizing COVID-19 in elderly patients. In this study, we presented the clinical features and outcomes estimated by overall survival in a cohort of elderly COVID-19 patients over 70 years old (yr).

## Methods

### Study design and patients

For this retrospective study, we enrolled consecutive hospitalized patients over 70 yr from Renmin Hospital of Wuhan University in Wuhan, China, from January 20 to February 15, 2020. All patients were followed up until March 15, 2020. Renmin Hospital of Wuhan University, which is located in the endemic areas of COVID-19, serves as an officially designated hospital. All the patients were diagnosed according to the clinical diagnosis standard by the WHO interim guidance, in which SARS-CoV-2 RNA was confirmed. The study was approved by the Renmin Hospital of Wuhan University Ethics Committee (approval number WDRY2020-K053). The ethical committee did not require informed consent from the patients since no direct contact with the patients occurred when the medical records were examined. According to the national and local policies, patients with COVID-19 and their relatives need to be isolated strictly. In addition, patients’ data were abstracted and recorded anonymously. Despite all this, patients or their relatives involved were asked for oral consent by telephone before enrollment. The oral consent was documented along with other data in the case report form when data were extracted.

In the case of adults with cognitive decline, oral consent was obtained from a legal guardian or representative of these participants instead.

### Data collection

The epidemiological, demographic, clinical, laboratory, treatment, and outcome information of patients was extracted from patients’ electronic medical records. Laboratory data were collected on admission. All data were independently checked by two physicians. To determine the symptom data that electronic medical records cannot provide, physicians in charge made a detailed inquiry about the history of present illness and recorded it.

Nasopharyngeal swabs were collected for extracting SARS-CoV-2 RNA from patients to confirm SARS-CoV-2 infection. The procedures were in accordance with a previous study [7]. A chest computed tomography (CT) scan was used to confirm the presence of pneumonia and the outcome of lesions. All cases were confirmed to be a SARS-CoV-2 infection. From symptoms of flu to ARDS, patients with COVID-19 can develop a range of illnesses of varying severity. According to the standard issued by the Nation Health Commission of the People’s Republic of China (7th edition) [9], patients were divided into 4 clinical subtypes, including mild, moderate, severe, and critical types (Additional file 1). The clinical manifestations of mild and moderate types are similar, and the treatment effect is definite. There are different degrees of respiratory dysfunction and higher mortality in severe or critical types. We combined the mild and moderate types as the “general type”. All patients enrolled in this study had definite outcomes (dead or discharged). The patients were classified into two groups for outcome evaluation: survivor and non-survivor groups.

### Statistical analysis

Continuous variables were described as the mean ± standard deviation (SD) or median (interquartile range, IQR), when appropriate. When the data were normally distributed, Student’s t-test was conducted to compare the mean values; otherwise, the Wilcoxon test was used. The Chi-square test was applied to compare the differences between groups when the data were categorical variables, which are presented as the number of cases (percentage). When the data were limited, the Fisher’s exact test was conducted. Univariable Cox proportional hazards regression was used to analyze the associations between individual indicators and the outcomes of disease by calculating the hazard ratios (HRs) and the corresponding 95% confidence intervals (95% CIs). Multivariable Cox proportional hazard regression was further conducted by using forward and backward stepwise selection with *P* values of 0.10 for the forward procedure and 0.05 for the backward procedure. The Kaplan-Meier method was used to develop survival curves, and log-rank test was applied to compare the survival curves between groups. Statistical analysis was performed using SAS 9.4 (SAS Institute Inc., Cary, North Carolina, USA). All analyses were two-sided, and *P* <  0.05 was regarded as statistically significant.

## Results

### Epidemiological and clinical features

A total of 166 patients over 70 yr with COVID-19 were hospitalized in our hospital from January 20 to February 15, 2020. Nineteen cases were excluded due to a lack of data on critical information or follow-up. In total, 147 laboratory-confirmed cases of SARS-CoV-2 infection were included in this study. The median age was 76 (IQR 72–81, ranging from 70 to 95) years. Among them, 85 patients (57.8%) were male. The median follow-up time was 25 (IQR 10.5–37) days. The demographic features showed that only 1 case (0.7%) was mild type, 88 cases (59.8%) were moderate type, 42 cases (28.6%) were severe type, and 16 cases (10.9%) were critical type.

The most common symptoms of COVID-19 in elderly patients were fever (81.0%), cough (59.9%), fatigue (42.2%), dyspnea (40.8%), and expectoration (32.0%). Diarrhea (10.2%), pharyngalgia (4.8%), nausea (4.1%), vomiting (4.1%), and myalgia (3.4%) were rare. The median of the period from the first symptom onset to admission was 10 (IQR 7–14) days. Regarding comorbidities, 18.4% had respiratory disease (chronic bronchitis, chronic pneumonia, bronchial asthma, tuberculosis, etc.), 53.7% had cardiovascular disease (hypertension, coronary heart disease, atrial fibrillation, etc.), 21.8% had endocrine system disease (diabetes, hyperthyroidism, etc.), 3.4% had tumor, 21.8% had previously undergone surgery, and 19.1% had other comorbidities, including cirrhosis, cerebral infarction, etc. The total case-fatality rate was 28.6% (42/147, including 23 males and 19 females with a ratio of 1.21:1). The case-fatality rate increased with age, 21.8% (22/101) in patients aged 70–79 yr, 38.1% (16/42) in patients aged 80–89 yr, and 100% (4/4) in patients aged over 90 yr.

Of the entire cohort, 104 patients were cured or obviously improved until March 15, 2020. The survivors were younger (75 vs. 79, *P* = 0.005) and consisted of more patients in general type (*P* = 0.001; Table 1).

**Table 1:** Baseline characteristics of elderly patients with COVID-19

	Total	Survivor	Non-survivor	P^*^
	***n*** = 147 (%)	***n*** = 105 (%)	***n*** = 42 (%)	
**Age**, yr (median, IQR)	76 (72–81)	75 (72–79)	79 (75–84)	0.005
Sex				0.714
Male	85 (57.8)	62 (59.1)	23 (54.8)	
Female	62 (42.2)	43 (40.9)	19 (45.2)	
Symptoms				
Fever	119 (81.0)	86 (81.9)	33 (78.6)	0.647
Cough	88 (59.9)	62 (59.1)	26 (61.9)	0.853
Dyspnea	60 (40.8)	38 (36.2)	22 (52.4)	0.094
Expectoration	47 (32.0)	31 (29.5)	16 (38.1)	0.333
Pharyngalgia	7 (4.8)	4 (3.8)	3 (7.1)	0.408
Nausea	6 (4.1)	4 (3.8)	2 (4.8)	0.999
Vomiting	6 (4.1)	3 (2.9)	3 (7.1)	0.354
Diarrhea	15 (10.2)	11 (10.5)	4 (9.5)	0.999
Fatigue	62 (42.2)	43 (41.0)	19 (45.2)	0.713
Myalgia	5 (3.4)	4 (3.8)	1 (2.4)	0.999
Comorbidity				
Respiratory diseases	27 (18.4)	16 (15.2)	11 (26.2)	0.157
Cardiovascular disease	79 (53.7)	55 (52.4)	24 (57.1)	0.715
Endocrine System	32 (21.8)	25 (23.8)	7 (16.7)	0.385
Surgery	32 (21.8)	26 (17.7)	6 (4.1)	0.191
Tumor	5 (3.4)	4 (3.8)	1 (2.4)	0.999
Other comorbidities	28 (19.1)	18 (17.1)	10 (23.8)	0.360
**Type**				0.001
General	89 (60.5)	73 (69.5)	16 (38.1)	
Severe	42 (28.6)	24 (22.9)	18 (42.9)	
Critical	16 (10.9)	8 (7.6)	8 (19.0)	

^*^P values were calculated by the Student’s t-test or Wilcoxon test for continuous variables, and the Chi-square test for categorical variables; otherwise the Fisher’s exact test was used when the data were limited. yr, years old; IQR, interquartile range. *P <  0.05

### Laboratory findings

Regarding laboratory findings at admission to the hospital (Table 2; Additional file 2), the levels of leukocytes (8.16 vs. 5.96, *P* = 0.003) and neutrophils (7.03 vs. 4.26, *P* <  0.001) increased significantly in the non-survivors. The level of lymphocytes (0.55 vs. 0.88, *P* <  0.001) decreased more significantly in the non-survivors. Elevated level of aspartate aminotransferase (AST; *P* = 0.007), lactic dehydrogenase (LDH; *P* <  0.001), creatine kinase (*P* = 0.034), creatinine (*P* = 0.033), and blood urea nitrogen (BUN; *P* = 0.001) were also observed in the non-survivors of COVID-19 patients. In addition, indicators of inflammation, bacterial infection and blood coagulation, including procalcitonin (0.166 ng/mL vs. 0.076 ng/mL, *P* <  0.001), C-reactive protein (CRP; 107.3 vs. 37.65, *P* <  0.001), and D-dimer (6.32 vs. 1.09, *P* <  0.001), showed a higher level in the non-survivors.

**Table 2:** Laboratory findings in elderly patients with COVID-19

	Total median (IQR)	Survivor median (IQR)	Non-survivor median (IQR)	P^*^
**Leukocytes** (10^9^/L)	6.40 (4.54–9.00)	5.96 (4.53–7.85)	8.16 (5.58–12.50)	0.003
**Lymphocytes** (10^9^/L)	0.84 (0.54–1.18)	0.88 (0.68–1.34)	0.55 (0.36–0.88)	< 0.001
**Neutrophils** (10^9^ /L)	4.90 (3.10–7.44)	4.26 (2.85–6.10)	7.03 (4.30–7.03)	< 0.001
ALT (U/L)	25 (18–40)	26 (18–42)	23 (18–35)	0.24
**AST** (U/L)	31 (21–46)	30 (21–41)	42.5 (26–57)	0.007
**LDH** (U/L)	309 (223–467)	263 (214–359)	478.5 (363–584)	< 0.001
**CK** (U/L)	63 (42–114)	60 (41–93)	78 (45–215)	0.034
Albumin (g/L)	34.26 ± 4.05	34.56 ± 4.04	33.51 ± 4.02	0.999
Globulin (g/L)	25.3 (22.0–28.4)	24.7 (21.8–28.3)	25.7 (22.5–29.0)	0.14
**Creatinine** (μmol/L)	68.0 (56.0–92.0)	66.0 (57.0–81.0)	81.0 (54.0–120.0)	0.033
**BUN** (mmol/L)	6.50 (4.85–9.55)	6.20 (4.70–7.90)	9.90 (6.10–16.45)	< 0.001
**CD3** (/μL)	455.5 (267–709)	558 (377–840)	244 (142–397)	< 0.001
**CD4** (/μL)	281 (165–484)	333 (212–559)	165 (96–267)	< 0.001
**CD8** (/μL)	145 (67–255)	191 (96–267)	64 (42–139)	< 0.001
**CD19** (/μL)	109 (60–163)	114 (68–183)	75 (42–138)	0.016
**CD16 + CD56** (/μL)	101 (59–210)	116 (72–247)	69 (39–118)	0.002
**CRP** (mg/L)	53.85 (15.25–94.10)	37.65 (11.40–70.80)	107.3 (55.2–188.0)	< 0.001
**D-dimer** (mg/L)	1.61 (0.68–5.96)	1.09 (0.50–3.26)	6.32 (2.37–17.44)	< 0.001
CD4/CD8	2.10 (1.39–3.14)	2.03 (1.39–3.16)	2.22 (1.30–3.03)	0.685
**Procalcitonin** (ng/mL)	0.093 (0.052–0.189)	0.076 (0.046–0.137)	0.166 (0.085–0.552)	< 0.001
C3 (g/L)	0.970 ± 0.200	0.989 ± 0.196	0.931 ± 0.201	0.835
C4 (g/L)	0.244 (0.186–0.310)	0.241 (0.187–0.301)	0.254 (0.152–0.328)	0.955
IgM (g/L)	0.822 (0.597–1.140)	0.779 (0.562–1.090)	1.030 (0.715–1.320)	0.059
IgG (g/L)	12.4 (10.4–15.0)	12.1 (9.5–15.0)	12.9 (11.2–14.9)	0.219
**IgA** (g/L)	2.70 (2.12–3.44)	2.59 (1.98–3.32)	3.19 (2.42–3.63)	0.036
**IgE** (IU/mL)	37.6 (18.3–98.7)	32.8 (18.3–92.4	70.3 (27.8–164.0)	0.046

*P values were calculated by the Student’s t-test or Wilcoxon test for continuous variables. ALT, alanine aminotransferase; AST, aspartate aminotransferase; CK, creatine kinase; CRP, C-reactive protein; LDH, lactate dehydrogenase; BUN, blood urea nitrogen; C3, Complement component 3; C4, Complement component 4; IgM, immunoglobulin M; IgG, immunoglobulin G; IgA, immunoglobulin A; IgE, immunoglobulin E; CD, cluster of differentiation. *P <  0.05

Compared to the survivors, levels of CD3, CD4, CD8, CD19, and CD16 + CD56 T cells were decreased significantly in the non-survivors, while the level of immunoglobulin (Ig) A and IgE were increased (Table 2). Differences in the other indicators of humoral immunity, including IgG, IgM, complement component 3 and 4, were not significant between the two groups (Table 2).

### Treatments in elderly patients with COVID-19

The main treatments included antiviral therapy (arbidol, oseltamivir, ribavirin, etc.; 97.3%), antimicrobial therapy (moxifloxacin, cefoperazone, meropenem, etc.; 84.4%), oxygen therapy (89.8%), and traditional Chinese medicine (73.5%). According to the individual’s health conditions, hormones (47.6%), gamma globulin (44.9%) and vasoactive drugs (21.8%) were also used as personalized medicine. At the same time, according to the comorbidities of COVID-19 patients, hemodialysis (2.6%) and other corresponding symptomatic support treatments, including transfusion of human albumin, nutrition support and so on, were provided (Table 3).

**Table 3:** Treatments in elderly patients with COVID-19

	Total	Survivor	Non-survivor	P^*^
	***n*** = 147 (%)	***n*** = 105 (%)	***n*** = 42 (%)	
Antivirals	143 (97.3)	104 (99.1)	39 (92.9)	0.071
**Antibiotics**	124 (84.4)	82 (78.1)	42 (100.0)	< 0.001
**Hormone**	70 (47.6)	42 (40.0)	28 (66.7)	0.006
Gamma globulin	66 (44.9)	43 (41.0)	23 (54.8)	0.145
Chinese medicine	108 (73.5)	81 (77.1)	27 (64.3)	0.147
Oxygen	132 (89.8)	91 (86.7)	41 (97.6)	0.068
**Vasoactive drugs**	32 (21.8)	3 (2.9)	29 (69.1)	< 0.001
Hemodialysis	4 (2.7)	3 (2.9)	1 (2.4)	0.999
Other therapy	95 (64.6)	64 (61.0)	31 (73.8)	0.182

^*^P values were calculated by the Chi-square test for categorical variables, and the Fisher’s exact test was used when the data were limited. *P <  0.05

As is shown in Table 3, antibiotic treatment (*P* <  0.001) and vasoactive drugs (*P* <  0.001) were used more often in the non-survivors.

### Predictors for death of elderly patients with COVID-19

**Fig. 1:**
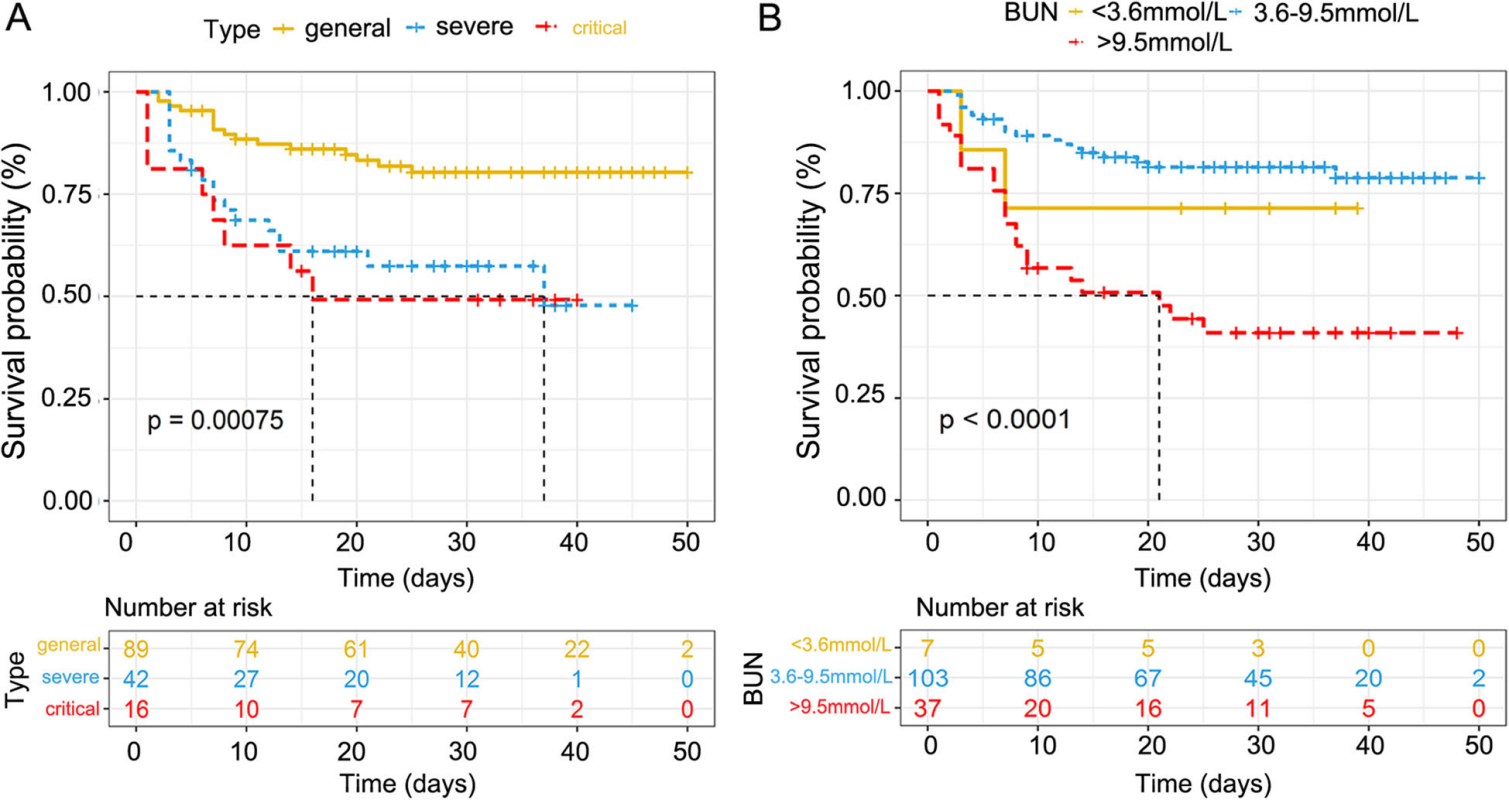
Survival curves for elderly COVID-19 patients with different severity of disease and blood urea nitrogen. A, Elderly patients in severe and critical type had higher risk of death when compared with the general type (*P* = 0.00075). B, Elderly patients with elevated level of BUN (> 9.5 mmol/L) had higher risk of death (*P* <  0.0001)

Kaplan-Meier curves indicated that severe and critical type, and elevated level of BUN increased the risk of death in elderly patients with COVID-19 (*P* = 0.00075 and <  0.0001 respectively by log-rank test; Fig. 1). Cox proportional hazards regression were performed to identify the risk factors that were associated with the outcomes of COVID-19 patients. As summarized in Table 4, factors including age, type, level of leukocytes, neutrophils, lymphocytes, AST, CK, BUN, LDH, procalcitonin, CD3, CD4, CD8, CD16 + CD56 T cells, hormone therapy, antiviral therapy, and vasoactive drugs were associated with the outcomes of elderly patients. In multivariable Cox proportional hazard regression analyses, clinical subtypes including the severe type (HR = 2.983, 95%CI: 1.231–7.226, *P* = 0.016), and the critical type (HR = 3.267, 95%CI: 1.009–10.576, *P* = 0.048) were associated with increasing risk of death when compared with the general type, and BUN greater than 9.5 mmol/L (HR = 2.805, 95% CI: 1.141–6.892, *P* = 0.025) on admission was the only risk factor for death among laboratory findings.

**Table 4:** Univariable and multivariable analyses of the association between clinical and laboratory variables and death

	Total n for analysis	Univariable Analysis HR (95%CI)	***P***	Multivariable Analysis HR (95%CI)	P^*^
Age	147	1.079 (1.027–1.134)	0.003		
Type	147				
**Severe**	42	2.896 (1.473–5.692)	0.002	2.983 (1.231–7.226)	0.016
**Critical**	16	3.542 (1.515–8.284)	0.004	3.267 (1.009–10.576)	0.048
Leukocytes (10^9^/L)	147				
< 3.5	13	3.153 (1.251–7.948)	0.015		
> 9.5	32	3.931 (2.043–7.563)	< 0.001		
Lymphocytes (10^9^/L)	147				
< 1.1	100	2.656 (1.179–5.983)	0.018		
Neutrophils (10^9^/L)	147				
< 1.8	9	1.289 (0.296–5.611)	0.735		
> 6.3	48	3.366 (1.786–6.344)	0.001		
AST (U/L)	147				
< 15	4	1.180 (0.158–8.831)	0.872		
> 40	50	2.523 (1.365–4.664)	0.003		
CK (U/L)	147				
< 50	50	0.814 (0.395–1.680)	0.578		
> 310	15	2.990 (1.373–6.511)	0.006		
Creatinine (μmol/L)	147				
< 57	37	1.527 (0.715–3.261)	0.274		
> 111	23	3.987 (1.961–8.103)	0.001		
BUN (mmol/L)	147				
< 3.6	7	1.623 (0.378–6.969)	0.515	3.812 (0.788–18.435)	0.096
**> 9.5**	37	3.913 (2.099–7.291)	< 0.001	2.805 (1.141–6.892)	0.025
Globulin (g/L)	147				
< 20	12	0.263 (0.036–1.915)	0.187		
> 40	3	2.042 (0.493–8.468)	0.325		
Dyspnea	147	1.641 (0.895–3.007)	0.109		
Hormone therapy	147	2.393 (1.260–4.547)	0.008		
Antiviral therapy	147	0.188 (0.057–0.615)	0.006		
Oxygen therapy	147	4.154 (0.571–30.210)	0.160		
**Vasoactive drugs**	147	14.126 (7.238–27.569)	< 0.001	16.120 (6.573–39.533)	< 0.001
LDH (U/L)	147				
> 250	98	5.221 (1.862–14.639)	0.002		
Procalcitonin (ng/mL)	137				
> 0.1	64	2.715 (1.405–5.244)	0.003		
CD3 (/μL)	122				
< 723	93	9.718 (1.325–71.263)	0.025		
CD4 (/μL)	122				
< 404	95	6.496 (1.550–27.232)	0.011		
CD8 (/μL)	122				
< 220	85	3.089 (1.081–8.829)	0.035		
CD19 (/μL)	122				
< 80	43	1.975 (0.976–3.995)	0.058		
CD16 + CD56 (/μL)	122				
< 84	49	2.516 (1.221–5.186)	0.012		
IgA (g/L)	121				
> 4.0	19	1.012 (0.388–2.635)	0.981		
IgM (g/L)	121				
< 0.4	14	0.946 (0.286–3.126)	0.927		
> 2.3	3	2.416 (0.573–10.184)	0.230		
IgE (IU/mL)	121				
> 100	30	1.541 (0.725–3.272)	0.261		

*A forward and backward stepwise selection with *P* values of 0.10 for the forward procedure and 0.05 for the backward procedure was used to select the variables for the final model. AST, aspartate aminotransferase; CK, creatine kinase; LDH, lactate dehydrogenase; BUN, blood urea nitrogen; IgM, immunoglobulin M; IgA, immunoglobulin A; IgE, immunoglobulin E; CD, cluster of differentiation. *P < 0.05

## Discussion

This report provides an insight into the clinical characteristics and risk factors associated with death in elderly patients over 70 yr with laboratory-confirmed COVID-19 from a single center in Wuhan, China. There are some important findings in this study. Clinical types and BUN levels greater than 9.5 mmol/L were associated with higher odds of death. In addition, several laboratory findings such as elevated levels of AST, LDH, CK, creatinine, procalcitonin, and D-dimer may help us evaluate the outcomes of critically ill patients.

The case-fatality rate was 28.6%, much higher than the reported total fatality rate of 2.3% [10]. The severity of disease was associated with the outcomes of patients. The patients of general type had a relatively lower case-fatality rate than that for the severe patients. As the severity of the disease escalated, the fatality rate obviously increased, from 18.0% in the general type to 42.9% in the severe type, and 50.0% in the critical type. Furthermore, survival analysis suggested that the severity of the disease was closely related to the prognosis. These results suggested that the classification of the severity of COVID-19 was reasonable and closely related to the adverse outcomes.

The duration between symptom onset and hospitalization was 10 days (range 7–14), which is slightly longer than that reports in other studies [6, 8]. This is reasonable because the hospitals in Wuhan, China had been overwhelmed by a flood of patients. The clinical manifestations of patients observed in this study were similar to those previously reported in terms of symptoms and frequency [6, 11, 12]. The common symptoms of COVID-19 were fever, cough, expectoration, pharyngalgia, dyspnea, nausea, etc., which presented no differences between the two groups. Moreover, diarrhea occurred in 10.2% of the patients. As an atypical manifestation, diarrhea presented as the only symptom without fever in some cases, which increased the difficulty in the diagnosis of COVID-19. However, the incidence of diarrhea in this disease was much lower than that in SARS or MERS, which was shown to be 20.3 and 22%, respectively [11, 13]. In our study, RNA of virus was all tested in nasopharyngeal swabs. It was reported that SARS-CoV-2 had been detected in stool, which suggested a possibility of fecal-oral transmission [14, 15]. As previously reported, underlying comorbidities including hypertension, cardiovascular disease, diabetes mellitus, cerebrovascular disease, were found to be associated with the outcomes of the disease [8, 16]. However, we did not find any comorbidity was associated with death. This may be partially explained by the higher proportion of underlying comorbidities in elderly patients. In addition, it was speculated that it is the severity of comorbidity rather than its presence that could affect the prognosis. The severity of comorbidity was also not rated in previous studies, which raised some uncertainty about these data.

In this study, we were able to identify some clinical and laboratory features at admission that were associated with the adverse clinical outcome of death. Elevated levels of AST, LDH, CK, creatinine, BUN, procalcitonin, and D-dimer, or decreased lymphocytes were more common in the patients with adverse outcomes. With further measurement of lymphocyte subsets, CD3, CD4, CD8, CD19 and CD16 + CD56 positive T cells were shown to be obviously decreased in the non-survivor group. As reported in MERS, T-cells were important in clearing virus [17]. These results suggested that the decrease in the T-cell number indicated an adverse outcome [18]. While in elderly patients, the activation of immune system is limited after severe immune injury, which may account for the deteriorated outcome [19]. A possible reason for the lymphopenia may be that lymphocytes are directly infected and destroyed by SARS-CoV-2, but this needs to be validated [20]. In addition, deficiency of antibody response indicated poor prognosis in MERS [21]. However, total level of IgG and IgM showed no significant change in patients with different outcomes in our study, which indicated that it is the response but not baseline of antibody that impact the outcome of patients. SARS-CoV-2-specific IgG and IgM need to be further evaluated during the progression of COVID-19. All the abnormalities suggest that SARS-CoV-2 infection may be associated with myocardia injury, hepatic injury, kidney injury, and cellular immune deficiency.

Notably, we found that only BUN greater more than 9.5 mmol/L in the laboratory findings was associated with adverse outcomes. These results reflected collinearity of other laboratory findings with the clinical subtype. As one of the key elements of pneumonia severity index (PSI), BUN is an important indicator to evaluate the severity of pneumonia and predict the outcomes of patients [22]. In SARS, elevated level of BUN implied the renal dysfunction for virus could be detected in epithelial cells of renal distal tubules [23]. SARS-CoV-2 was detected in urine samples in other studies [14, 24]. BUN is an indicator of renal insufficiency. Compared to SARS, MERS progress more rapidly to acute kidney injury [25]. In this study, one possible explanation is that the SARS-CoV-2 infection causes the inflammatory storm. The generation of cytokine storm can lead to acute kidney injury, which is a nonnegligible cause of death. In addition, elevated level of BUN was observed to be the independent predictor of bacteremia in community-acquired pneumonia [26]. Combined with the increased level of leukocyte and procalcitonin in non-survivors of elderly patients with COVID-19, particular attention should be paid on the abnormal level of BUN.

According to the previous coronavirus infection and clinical cognition, there is no specific treatment for the infection, mainly limited to support organ functions. Antiviral therapy is partially effective, primarily to delay the progress of the disease and restore autoimmune function [27]. Antibiotic therapy may be needed in elderly patients with basic pulmonary conditions. It’s remarkable that patients who received vasoactive drugs treatment were more likely to develop adverse outcomes, which may be confounded by indication. Specially, critical patients were more likely to be given vasoactive drugs. However, due to potential bias and confounding factors, these results should be interpreted with caution. Further study should be conducted to find out the potential factors.

Nevertheless, the present study had some limitations. First, patients with false-negative nasopharyngeal swabs were not hospitalized in time. In addition, patients with COVID-19 in our hospital were relatively serious. These factors may lead to high adverse outcomes. Second, the number of patients over 70 yr was relatively small, which is partly related to the less social contact of the elderly. Elderly patients are more likely to delay being admitted to the hospital. Considering these reasons, we need a larger cohort study to draw more accurate conclusions. Third, some important information such as patients’ weight, BMI, and information to assess the severity of comorbidities were unavailable at the outbreak of epidemic, for data was extracted from patients’ electronic medical records. Finally, at the time of the outbreak of acute infectious diseases, these cases are not the natural state of the disease. These data may be more convincing when the epidemic is over.

## Conclusion

The patients over 70 yr with COVID-19 had a high case-fatality rate. The severity of the disease was closely related to the prognosis. Elevated BUN is an independent risk factor for death.

## Supplementary Information

**Additional file 1.** Clinical Classification of COVID-19 Patients.

**Additional file 2.** Laboratory findings in elderly patients with COVID-19.

## Data Availability

All data analyzed during this study are included in this published article. The raw datasets used for the analysis are available from the corresponding author on reasonable request.

## Abbreviations

SARS-CoV-2: Severe acute respiratory syndrome coronavirus-2
WHO: World Health Organization
COVID-19: 2019 novel coronavirus disease
MERS-CoV: Middle East respiratory syndrome coronavirus
NCIP: Novel coronavirus-infected pneumonia
ARDS: Acute respiratory distress syndrome.
yr: years old
CT: Computed tomography
SD: Standard deviation
IQR: Interquartile range
HRs: Hazard ratios
95% CIs: 95% confidence intervals
AST: Aspartate aminotransferase
LDH: Lactic dehydrogenase
BUN: Blood urea nitrogen
CRP: C-reactive protein
Ig: Immunoglobulin
