# Supplementary material for: Risk factors for mortality in patients over 70 years old with COVID-19 in Wuhan at the early break: retrospective case series
Source: BMC Infect Dis. 2021 Aug 16;21:821. doi: 10.1186/s12879-021-06450-8 (PMC8366151; doi:10.1186/s12879-021-06450-8)
Supplement: Supplementary file 1 — Additional file 1. Clinical Classification of COVID-19 Patients. [file 12879_2021_6450_MOESM1_ESM.docx]

**Additional file 1.** **Clinical Classification of COVID-19 Patients**

1. Mild cases

The clinical symptoms were mild, and there was no sign of pneumonia on imaging.

2. Moderate cases

Showing fever and respiratory symptoms with radiological findings of pneumonia.

3. Severe cases

Adult cases meeting any of the following criteria:

3.1 Respiratory distress (≥ 30 breaths / min);

3.2 Oxygen saturation ≤ 93% at rest;

3.3 Arterial partial pressure of oxygen (PaO2) / fraction of inspired oxygen (FiO2) ≤ 300mmHg (l mmHg = 0.133kPa).

In high-altitude areas (at an altitude of over 1,000 meters above the sea level), PaO2/ FiO2 shall be corrected by the following formula: PaO2 / FiO2 x[Atmospheric pressure (mmHg) / 760]

Cases with chest imaging that shows obvious lesion progression within 24-48 hours >50% shall be managed as severe cases.

4. Critical cases

Cases meeting any of the following criteria:

4.1 Respiratory failure and requiring mechanical ventilation;

4.2 Shock;

4.3 With other organ failure that requires ICU care.
