# Supplementary material for: Risk factors for mortality in patients over 70 years old with COVID-19 in Wuhan at the early break: retrospective case series
Source: BMC Infect Dis. 2021 Aug 16;21:821. doi: 10.1186/s12879-021-06450-8 (PMC8366151; doi:10.1186/s12879-021-06450-8)
Supplement: Supplementary file 2 — Additional file 2. Laboratory findings in elderly patients with COVID-19. [file 12879_2021_6450_MOESM2_ESM.docx]

| **Additional file 2. Laboratory findings in elderly patients with COVID-19** | | | | |
| --- | --- | --- | --- | --- |
|  | **Total** | **Survivor** | **Non-survivor** | ***P^*^*** |
| **Leukocyte** (10^9^/L) |  |  |  | <0.001 |
| <3.5, n (%) | 13 (8.8) | 7 (6.7) | 6 (14.2) |  |
| 3.5-9.5, n (%) | 102 (69.4) | 84 (80.0) | 18 (42.9) |  |
| >9.5, n (%) | 32 (21.8) | 14 (13.3) | 18 (42.9) |  |
| **Lymphocytes** (10^9^/L) |  |  |  | 0.012 |
| <1.1, n (%) | 100 (68.0) | 65 (61.9) | 35 (83.3) |  |
| 1.1-3.2, n (%) | 47 (32.0) | 40 (38.1) | 7 (16.7) |  |
| **Neutrophils** (10^9^ /L) |  |  |  | <0.001 |
| <1.8, n (%) | 9 (6.1) | 7 (6.7) | 2 (4.8) |  |
| 1.8-6.3, n (%) | 90 (61.2) | 74 (70.5) | 16 (38.1) |  |
| >6.3, n (%) | 48 (32.7) | 24 (22.8) | 24 (57.1) |  |
| ALT (U/L) |  |  |  | 0.239 |
| <9, n (%) | 3 (2.0) | 1 (1.0) | 2 (4.8) |  |
| 9-50, n (%) | 124 (84.4) | 88 (83.8) | 36 (85.7) |  |
| >50, n (%) | 20 (13.6) | 16 (15.2) | 4 (9.5) |  |
| **AST** (U/L) |  |  |  | 0.012 |
| <15, n(%) | 4 (2.7) | 3 (2.8) | 1 (2.4) |  |
| 15-40, n (%) | 93 (63.3) | 74 (70.5) | 19 (45.2) |  |
| >40, n (%) | 50 (34.0) | 28 (26.7) | 22 (52.4) |  |
| **LDH** (U/L) |  |  |  | < 0.001 |
| 120-250, n (%) | 49 (33.3) | 45 (42.9) | 4 (9.5) |  |
| >250, n (%) | 98 (66.7) | 60 (57.1) | 38 (90.5) |  |
| **CK** (U/L) |  |  |  | 0.015 |
| <50, n (%) | 50 (34.0) | 39 (37.2) | 11 (26.2) |  |
| 50-310, n (%) | 82 (55.8) | 60 (57.1) | 22 (52.4) |  |
| >310, n (%) | 15 (10.2) | 6 (5.7) | 9 (21.4) |  |
| Albumin (g/L) |  |  |  | 0.282 |
| <40, n (%) | 137 (93.2) | 96 (91.4) | 41 (97.6) |  |
| 40-55, n (%) | 10 (6.8) | 9 (8.6) | 1(2.4) |  |
| Globulin (g/L) |  |  |  | 0.100 |
| <20, n (%) | 12 (8.2) | 11 (10.5) | 1 (2.4) |  |
| 20-40, n (%) | 132 (89.8) | 93 (88.5) | 39 (92.9) |  |
| >40, n (%) | 3 (2.0) | 1 (1.0) | 2 (4.76) |  |
| **Creatinine** (μmol/L) |  |  |  | <0.001 |
| <57, n(%) | 37 (25.2) | 26 (24.8) | 11 (26.2) |  |
| 57-111, n (%) | 87 (59.2) | 70 (66.7) | 17 (40.5) |  |
| >111, n (%) | 23 (15.6) | 9 (8.6) | 14 (33.3) |  |
| **BUN** (mmol/L) |  |  |  | <0.001 |
| <3.6 | 7 (4.8) | 5 (4.8) | 2 (4.8) |  |
| 3.6-9.5, n (%) | 103 (70.1) | 84 (80.0) | 19 (45.2) |  |
| >9.5, n (%) | 37 (25.2) | 16 (15.2) | 21 (50.0) |  |
| **CD3** (/μL) |  |  |  | 0.001 |
| <723, n (%) | 93 (76.2) | 63 (69.2) | 30 (96.8) |  |
| 723-2737, n (%) | 29 (23.8) | 28 (30.8) | 1 (3.2) |  |
| **CD4** (/μL) |  |  |  | 0.001 |
| <404, n (%) | 95 (69.7) | 56 (61.5) | 29 (93.6) |  |
| 404-1612, n (%) | 37 (30.3) | 35 (38.5) | 2 (6.5) |  |
| **CD8** (/μL) |  |  |  | 0.014 |
| <220, n (%) | 85 (69.7) | 58 (63.7) | 27 (87.1) |  |
| 220-1129, n (%) | 37 (30.3) | 33 (36.3) | 4 (12.9) |  |
| CD19 (/μL) |  |  |  | 0.079 |
| <80, n (%) | 43 (35.3) | 27 (29.7) | 16 (51.6) |  |
| 80-616 n (%) | 78 (63.9) | 63 (69.2) | 15 (48.4) |  |
| >616, n(%) | 1 (0.8) | 1 (1.1) | 0 (0) |  |
| **CD16+CD56** (/μL) |  |  |  | 0.010 |
| <84, n (%) | 49 (40.2) | 30 (33.0) | 19 (61.3) |  |
| 84-724, n (%) | 73 (59.8) | 61 (67.0) | 12 (38.7) |  |
| **CRP** (mg/L) |  |  |  | <0.001 |
| 0-10, n (%) | 24 (17.7) | 24 (24.5) | 0 (0) |  |
| >10, n (%) | 112 (82.3) | 74 (75.5) | 38 (100.0) |  |
| **D-dimer** (mg/L) |  |  |  | <0.001 |
| 0-0.55, n (%) | 27 (19.3) | 27 (27.0) | 0 (0) |  |
| >0.55, n (%) | 113 (80.7) | 73 (73.0) | 40 (100.0) |  |
| CD4/CD8 |  |  |  | 0.746 |
| <0.9, n (%) | 15 (12.3) | 12 (13.2) | 3 (9.7) |  |
| 0.9-2.0, n (%) | 43 (35.3) | 33 (36.3) | 10 (32.3) |  |
| >2.0, n (%) | 64 (52.4) | 46 (50.5) | 18 (58.0) |  |
| **Procalcitonin** (ng/mL) |  |  |  | 0.001 |
| <0.1, n (%) | 73 (53.3) | 60 (62.5) | 13 (31.7) |  |
| ≥0.1, n (%) | 64 (46.7) | 36 (37.5) | 28 (68.3) |  |
| C3 (g/L) |  |  |  | 0.670 |
| <0.9, n(%) | 46 (38.0) | 33 (36.7) | 13 (41.9) |  |
| 0.9-1.8,n(%) | 75 (62.0) | 57 (63.3) | 18 (58.1) |  |
| C4 (g/L) |  |  |  | 0.182 |
| <0.1, n(%) | 10 (8.3) | 5(5.6) | 5 (16.1) |  |
| 0.1-0.4, n(%) | 103 (85.1) | 79 (87.8) | 24 (77.4) |  |
| >0.4 ,n(%) | 8 (6.6) | 6 (6.7) | 2 (6.5) |  |
| IgM (g/L) |  |  |  | 0.246 |
| <0.4, n(%) | 14 (11.6) | 11 (12.2) | 3 (9.7) |  |
| 0.4-2.3, n(%) | 104 (85.9) | 78 (86.7) | 26 (83.9) |  |
| >2.3, n(%) | 3 (2.5) | 1 (1.1) | 2 (6.4) |  |
| IgG (g/L) |  |  |  | 0.742 |
| <7, n(%) | 1 (0.8) | 1 (1.1) | 0 (0) |  |
| 7-16, n(%) | 97 (80.2) | 71 (78.9) | 26 (83.9) |  |
| >16, n(%) | 23 (19.0) | 18 (20.0) | 5 (16.1) |  |
| IgA (g/L) |  |  |  | 0.999 |
| 0.7-4.0, n(%) | 102 (84.3) | 76 (84.4) | 26 (83.9) |  |
| >4.0, n(%) | 19 (15.7) | 14 (15.6) | 5 (16.1) |  |
| IgE (IU/mL) |  |  |  | 0.335 |
| <100, n(%) | 91 (75.2) | 70 (77.8) | 21 (67.7) |  |
| ≥100, n(%) | 30 (24.8) | 21 (22.2) | 10 (32.3) |  |

**P* values were calculated by the Chi-square test for categorical variables; otherwise, the Fisher’s exact test was used when the data were limited. ALT, alanine aminotransferase; AST, aspartate aminotransferase; CK, creatine kinase; CRP, C-reactive protein; LDH, lactate dehydrogenase; BUN, blood urea nitrogen; LDH, lactic dehydrogenase; C3, Complement component 3; C4, Complement component 4; IgM, immunoglobulin M; IgG, immunoglobulin G; IgA, immunoglobulin A; IgE, immunoglobulin E; CD, cluster of differentiation.
